# 2,2′-Bis(all­yloxy)-1,1′-binaphth­yl

**DOI:** 10.1107/S1600536809015815

**Published:** 2009-04-30

**Authors:** Jia-Zhen Ge, Hui Li

**Affiliations:** a Ordered Matter Science Research Center, Southeast University, Nanjing 210096, People’s Republic of China

## Abstract

The complete mol­ecule of the title compound, C_26_H_22_O_2_, is generated by a crystallographic twofold rotation axis. The dihedral angle between the planes of the two symmetry-related naphthalene ring systems is 69.05 (4)°, while that between the naphthalene ring system and the allyl plane is 13.7 (2)°. No hydrogen bonds or aromatic π–π stacking inter­actions are observed.

## Related literature

For related structures, see: Fu & Zhao (2007 ▶); Zhang *et al.* (2008 ▶).
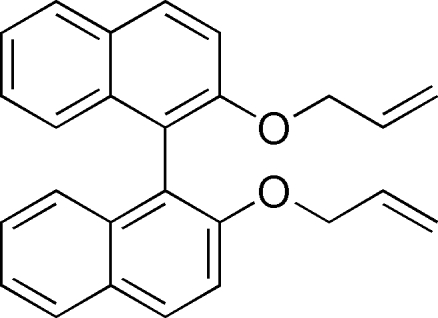


## Experimental

### 

#### Crystal data

C_26_H_22_O_2_
*M* *_r_* = 366.46
Tetragonal, 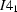

*a* = 11.7167 (9) Å
*c* = 14.583 (2) Å
*V* = 2001.9 (4) Å^3^
*Z* = 4
Mo *K*α radiation
μ = 0.08 mm^−1^
*T* = 298 K
0.20 × 0.18 × 0.14 mm

#### Data collection

Rigaku SCXmini diffractometer
Absorption correction: multi-scan (*CrystalClear*; Rigaku, 2005 ▶) *T*_min_ = 0.892, *T*_max_ = 0.990
5346 measured reflections
1024 independent reflections
806 reflections with *I* > 2σ(*I*)
*R*_int_ = 0.045

#### Refinement

*R*[*F* ^2^ > 2σ(*F* ^2^)] = 0.037
*wR*(*F* ^2^) = 0.095
*S* = 1.02
1024 reflections
127 parameters
1 restraint
H-atom parameters constrained
Δρ_max_ = 0.14 e Å^−3^
Δρ_min_ = −0.12 e Å^−3^

### 

Data collection: *CrystalClear* (Rigaku, 2005 ▶); cell refinement: *CrystalClear*; data reduction: *CrystalClear*; program(s) used to solve structure: *SHELXS97* (Sheldrick, 2008 ▶); program(s) used to refine structure: *SHELXL97* (Sheldrick, 2008 ▶); molecular graphics: *SHELXTL* (Sheldrick, 2008 ▶); software used to prepare material for publication: *SHELXTL*.

## Supplementary Material

Crystal structure: contains datablocks I, global. DOI: 10.1107/S1600536809015815/ci2788sup1.cif

Structure factors: contains datablocks I. DOI: 10.1107/S1600536809015815/ci2788Isup2.hkl

Additional supplementary materials:  crystallographic information; 3D view; checkCIF report

## Figures and Tables

**Fig. 1.**
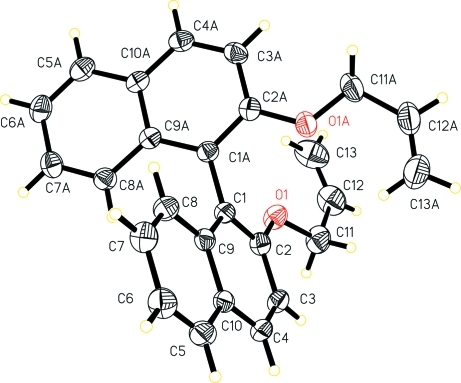
The molecular structure of the compound, with the atomic numbering scheme. Displacement ellipsoids are drawn at the 30% probability level. Atoms labelled with the suffix A are generated by the symmetry operation (1-x, -y, z).

## supplementary crystallographic information

### Comment

The molecule is located on a twofold rotation axis. The dihedral angle between the two naphthalene ring systems is 69.05 (4)° while that between the naphthalene ring and allyl plane is 13.7 (2)°. The molecule is twisted around the central C1—C1A bond with a torsion angle C2—C1—C1A—C2A of -66.6 (3)°. There are no remarkable short intermolecular interactions observed in the structure.

### Experimental

Racemic 1,1'-binaphthyl-2,2'-diol (2.86 g, 10 mmol) and allyl bromide (2.42 g, 20 mmol) were dissolved in acetone (50 ml) in the presence of K_2_CO_3_ (1.38 g, 10 mmol) and refluxed for 24 h. After the mixture was cooled to room temperature, the solution was filtered and rotated in vacuum. The title compound was purified by column chromatography with dichloromethane as eluent and was recrystallized from dichloromethane. Colorless single crystals of the title compound suitable for X-ray diffraction were obtained from an ethanol solution after a week.

### Refinement

H atoms were positioned geometrically and were allowed to ride on the C atoms to which they are bonded, with C-H = 0.93-0.97 Å and *U*_iso_(H) = 1.2*U*_eq_(C). In the absence of significant anomalous scattering, Friedel pairs were merged prior to the final refinement.

### Crystal data

**Table:**

C_26_H_22_O_2_	*D*_x_ = 1.216 Mg m^−^^3^
*M**_r_* = 366.46	Mo *K*α radiation, λ = 0.71073 Å
Tetragonal, *I*4_1_	Cell parameters from 1024 reflections
Hall symbol: I 4bw	θ = 2.0–27.5°
*a* = 11.7167 (9) Å	µ = 0.08 mm^−^^1^
*c* = 14.583 (2) Å	*T* = 298 K
*V* = 2001.9 (4) Å^3^	Prism, colourless
*Z* = 4	0.20 × 0.18 × 0.14 mm
*F*(000) = 776	

### Data collection

**Table:**

Rigaku SCXmini diffractometer	1024 independent reflections
Radiation source: fine-focus sealed tube	806 reflections with *I* > 2σ(*I*)
graphite	*R*_int_ = 0.045
Detector resolution: 13.6612 pixels mm^-1^	θ_max_ = 26.0°, θ_min_ = 2.2°
ω scans	*h* = −6→14
Absorption correction: multi-scan (*CrystalClear*; Rigaku, 2005)	*k* = −14→14
*T*_min_ = 0.892, *T*_max_ = 0.990	*l* = −17→17
5346 measured reflections	

### Refinement

**Table:**

Refinement on *F*^2^	Primary atom site location: structure-invariant direct methods
Least-squares matrix: full	Secondary atom site location: difference Fourier map
*R*[*F*^2^ > 2σ(*F*^2^)] = 0.037	Hydrogen site location: inferred from neighbouring sites
*wR*(*F*^2^) = 0.095	H-atom parameters constrained
*S* = 1.02	*w* = 1/[σ^2^(*F*_o_^2^) + (0.0479*P*)^2^ + 0.119*P*] where *P* = (*F*_o_^2^ + 2*F*_c_^2^)/3
1024 reflections	(Δ/σ)_max_ = 0.001
127 parameters	Δρ_max_ = 0.14 e Å^−^^3^
1 restraint	Δρ_min_ = −0.12 e Å^−^^3^

### Special details

**Table:**

Geometry. All e.s.d.'s (except the e.s.d. in the dihedral angle between two l.s. planes) are estimated using the full covariance matrix. The cell e.s.d.'s are taken into account individually in the estimation of e.s.d.'s in distances, angles and torsion angles; correlations between e.s.d.'s in cell parameters are only used when they are defined by crystal symmetry. An approximate (isotropic) treatment of cell e.s.d.'s is used for estimating e.s.d.'s involving l.s. planes.
Refinement. Refinement of *F*^2^ against ALL reflections. The weighted *R*-factor *wR* and goodness of fit *S* are based on *F*^2^, conventional *R*-factors *R* are based on *F*, with *F* set to zero for negative *F*^2^. The threshold expression of *F*^2^ > σ(*F*^2^) is used only for calculating *R*-factors(gt) *etc*. and is not relevant to the choice of reflections for refinement. *R*-factors based on *F*^2^ are statistically about twice as large as those based on *F*, and *R*- factors based on ALL data will be even larger.

### Fractional atomic coordinates and isotropic or equivalent isotropic displacement parameters (Å^2^)

**Table:**

	*x*	*y*	*z*	*U*_iso_*/*U*_eq_	
C9	0.3589 (2)	0.0005 (2)	0.65091 (16)	0.0410 (6)	
C1	0.4419 (2)	−0.0269 (2)	0.71920 (16)	0.0406 (6)	
C10	0.2494 (2)	−0.0516 (2)	0.65478 (17)	0.0419 (6)	
O1	0.49556 (16)	−0.12473 (17)	0.85183 (13)	0.0557 (5)	
C7	0.2987 (2)	0.1036 (2)	0.51508 (19)	0.0571 (8)	
H7A	0.3147	0.1559	0.4688	0.068*	
C2	0.4132 (2)	−0.1021 (2)	0.78793 (19)	0.0451 (6)	
C4	0.2253 (2)	−0.1283 (2)	0.72628 (18)	0.0490 (6)	
H4A	0.1537	−0.1624	0.7293	0.059*	
C8	0.3794 (2)	0.0793 (2)	0.57953 (19)	0.0486 (6)	
H8A	0.4499	0.1155	0.5764	0.058*	
C3	0.3047 (2)	−0.1536 (2)	0.79115 (19)	0.0499 (7)	
H3A	0.2873	−0.2049	0.8377	0.060*	
C5	0.1674 (2)	−0.0254 (3)	0.5868 (2)	0.0540 (7)	
H5A	0.0961	−0.0602	0.5888	0.065*	
C6	0.1911 (2)	0.0498 (3)	0.5184 (2)	0.0597 (8)	
H6A	0.1366	0.0658	0.4738	0.072*	
C11	0.4636 (3)	−0.1789 (3)	0.9343 (2)	0.0687 (9)	
H11A	0.4433	−0.2578	0.9224	0.082*	
H11B	0.3979	−0.1408	0.9607	0.082*	
C12	0.5612 (4)	−0.1738 (3)	0.9988 (3)	0.0834 (11)	
H12A	0.5519	−0.2102	1.0550	0.100*	
C13	0.6573 (4)	−0.1243 (4)	0.9849 (3)	0.0976 (13)	
H13C	0.6709	−0.0868	0.9298	0.117*	
H13A	0.7135	−0.1259	1.0300	0.117*	

### Atomic displacement parameters (Å^2^)

**Table:**

	*U*^11^	*U*^22^	*U*^33^	*U*^12^	*U*^13^	*U*^23^
C9	0.0387 (14)	0.0427 (14)	0.0415 (14)	0.0017 (11)	0.0029 (11)	−0.0071 (12)
C1	0.0377 (13)	0.0424 (14)	0.0419 (13)	−0.0010 (11)	0.0017 (12)	−0.0031 (12)
C10	0.0335 (14)	0.0452 (14)	0.0470 (14)	0.0019 (12)	0.0042 (12)	−0.0120 (12)
O1	0.0517 (11)	0.0685 (13)	0.0468 (10)	−0.0008 (10)	−0.0022 (9)	0.0134 (10)
C7	0.0595 (18)	0.0593 (18)	0.0524 (17)	0.0089 (15)	−0.0030 (14)	0.0075 (14)
C2	0.0455 (14)	0.0467 (14)	0.0430 (13)	0.0033 (12)	0.0010 (13)	−0.0022 (12)
C4	0.0389 (14)	0.0541 (15)	0.0539 (15)	−0.0053 (13)	0.0104 (13)	−0.0073 (13)
C8	0.0422 (15)	0.0506 (15)	0.0529 (15)	0.0006 (12)	0.0003 (13)	0.0025 (13)
C3	0.0504 (15)	0.0525 (15)	0.0468 (14)	−0.0058 (13)	0.0108 (14)	0.0037 (13)
C5	0.0372 (15)	0.0618 (17)	0.0630 (18)	0.0028 (13)	−0.0016 (14)	−0.0109 (16)
C6	0.0495 (17)	0.074 (2)	0.0556 (17)	0.0094 (15)	−0.0096 (15)	−0.0009 (16)
C11	0.085 (2)	0.073 (2)	0.0484 (17)	−0.0002 (18)	0.0014 (17)	0.0169 (16)
C12	0.106 (3)	0.085 (3)	0.0588 (19)	0.004 (2)	−0.020 (2)	0.0141 (19)
C13	0.106 (3)	0.089 (3)	0.098 (3)	0.001 (3)	−0.044 (3)	0.005 (3)

### Geometric parameters (Å, °)

**Table:**

C9—C8	1.411 (4)	C4—H4A	0.93
C9—C10	1.422 (3)	C8—H8A	0.93
C9—C1	1.428 (3)	C3—H3A	0.93
C1—C2	1.376 (3)	C5—C6	1.360 (4)
C1—C1^i^	1.500 (5)	C5—H5A	0.93
C10—C4	1.405 (4)	C6—H6A	0.93
C10—C5	1.415 (4)	C11—C12	1.482 (5)
O1—C2	1.368 (3)	C11—H11A	0.97
O1—C11	1.411 (4)	C11—H11B	0.97
C7—C8	1.363 (4)	C12—C13	1.282 (5)
C7—C6	1.410 (4)	C12—H12A	0.93
C7—H7A	0.93	C13—H13C	0.93
C2—C3	1.408 (4)	C13—H13A	0.93
C4—C3	1.359 (4)		
			
C8—C9—C10	117.6 (2)	C4—C3—C2	120.1 (3)
C8—C9—C1	123.1 (2)	C4—C3—H3A	120.0
C10—C9—C1	119.3 (2)	C2—C3—H3A	120.0
C2—C1—C9	119.0 (2)	C6—C5—C10	121.0 (3)
C2—C1—C1^i^	119.4 (2)	C6—C5—H5A	119.5
C9—C1—C1^i^	121.6 (2)	C10—C5—H5A	119.5
C4—C10—C5	121.5 (2)	C5—C6—C7	119.8 (3)
C4—C10—C9	119.0 (2)	C5—C6—H6A	120.1
C5—C10—C9	119.5 (2)	C7—C6—H6A	120.1
C2—O1—C11	118.8 (2)	O1—C11—C12	108.6 (3)
C8—C7—C6	120.2 (3)	O1—C11—H11A	110.0
C8—C7—H7A	119.9	C12—C11—H11A	110.0
C6—C7—H7A	119.9	O1—C11—H11B	110.0
O1—C2—C1	116.6 (2)	C12—C11—H11B	110.0
O1—C2—C3	122.1 (2)	H11A—C11—H11B	108.4
C1—C2—C3	121.3 (2)	C13—C12—C11	126.6 (4)
C3—C4—C10	121.2 (2)	C13—C12—H12A	116.7
C3—C4—H4A	119.4	C11—C12—H12A	116.7
C10—C4—H4A	119.4	C12—C13—H13C	120.0
C7—C8—C9	121.8 (3)	C12—C13—H13A	120.0
C7—C8—H8A	119.1	H13C—C13—H13A	120.0
C9—C8—H8A	119.1		
			
C8—C9—C1—C2	178.0 (2)	C5—C10—C4—C3	179.6 (2)
C10—C9—C1—C2	−0.9 (3)	C9—C10—C4—C3	−0.2 (4)
C8—C9—C1—C1^i^	−0.2 (4)	C6—C7—C8—C9	0.0 (4)
C10—C9—C1—C1^i^	−179.1 (2)	C10—C9—C8—C7	−1.3 (4)
C8—C9—C10—C4	−178.5 (2)	C1—C9—C8—C7	179.8 (2)
C1—C9—C10—C4	0.4 (3)	C10—C4—C3—C2	0.4 (4)
C8—C9—C10—C5	1.7 (3)	O1—C2—C3—C4	179.8 (2)
C1—C9—C10—C5	−179.4 (2)	C1—C2—C3—C4	−0.9 (4)
C11—O1—C2—C1	165.4 (2)	C4—C10—C5—C6	179.3 (3)
C11—O1—C2—C3	−15.3 (4)	C9—C10—C5—C6	−0.8 (4)
C9—C1—C2—O1	−179.6 (2)	C10—C5—C6—C7	−0.5 (4)
C1^i^—C1—C2—O1	−1.4 (4)	C8—C7—C6—C5	0.9 (4)
C9—C1—C2—C3	1.1 (3)	C2—O1—C11—C12	−169.1 (2)
C1^i^—C1—C2—C3	179.3 (3)	O1—C11—C12—C13	3.0 (5)

Symmetry codes: (i) −*x*+1, −*y*, *z*.
